# Improving Clinical Decision-Making in Treating Airway Diseases With an Expert System Built Upon the Free AI Tool Google NotebookLM

**DOI:** 10.2196/78567

**Published:** 2026-01-29

**Authors:** Cheng-Hao Hsu, Ching-Li Hsu, Chih-Hsiang Tsou, Kuo-Fang Hsu, Hung-Yu Yang

**Affiliations:** 1 Department of Medicine College of Medicine Taipei Medical University Taipei Taiwan; 2 Miaoli General Hospital, Ministry of Health and Welfare of Taiwan Miaoli City Taiwan; 3 Department of Pulmonology Jen-Ai Branch, Taipei City Hospital Taipei Taiwan; 4 School of Medicine College of Medicine National Yang Ming Chiao Tung University Taipei Taiwan; 5 Department of Cardiology Wan Fang Hospital Taipei Medical University Taipei Taiwan; 6 Biomedical Data Center Wan Fang Hospital Taipei Medical University Taipei Taiwan

**Keywords:** airway disease, artificial intelligence, AI, artificial intelligence tool, AI tool, emergency department, medical decision-making, NotebookLM, pulmonology

## Abstract

We used the free artificial intelligence (AI) tool Google NotebookLM, powered by the large language model Gemini 2.0, to construct a medical decision-making aid for diagnosing and managing airway diseases and subsequently evaluated its functionality and performance in a clinical workflow. After feeding this tool with relevant published clinical guidelines for these diseases, we evaluated the feasibility of the system regarding its behavior, ability, and potential, and we created simulated cases and used the system to solve associated medical problems. The test and simulation questions were designed by a pulmonologist, and the appropriateness (focusing on accuracy and completeness) of AI responses was judged by 3 pulmonologists independently. The system was then deployed in an emergency department setting, where it was tested by medical staff (n=20) to assess how it affected the process of clinical consultation. Test opinions were collected through a questionnaire. Most (56/84, 67%) of the specialists’ ratings regarding AI responses were above average. The interrater reliability was moderate for accuracy (intraclass correlation coefficient=0.612; *P*<.001) and good on completeness (intraclass correlation coefficient=0.773; *P*<.001). When deployed in an emergency department (ED) setting, this system could respond with reasonable answers, enhance the literacy of personnel about these diseases. The potential to save the time spent in consultation did not reach statistical significance (Kolmogorov-Smirnov [K-S] D=0.223, *P*=.24) across all participants, but it indicated a favorable outcome when we analyzed only physicians’ responses. We concluded that this system is customizable, cost efficient, and accessible to clinicians and allied health care professionals without any computer coding experience in treating airway diseases. It provides convincing guideline-based recommendations, increases the staff’s medical literacy, and potentially saves physicians’ time spent on consultation. This system warrants further evaluation in other medical disciplines and health care environments.

## Introduction

### Expert Systems and the Decision-Making Aids

An expert system is a knowledge management tool that integrates domain-specific information with an inference engine to facilitate decision-making. When consulted, it delivers timely and relevant solutions to a problem. This concept originated in the 1950s, with medical diagnostic reasoning being among its earliest applications [1,2]. Gradually, expert systems became popular in the 1970s and 1980s [3]. With the advent of the digital era, the concept of expert systems was revisited by the medical community. For instance, oncology, given its rapid evolution and growth in knowledge over the past 2 decades, has required consultation systems to aid patient management.

### Knowledge Management and the Large Language Model

Large language models (LLMs) represent a branch of machine learning that has been under development for several years. Their impact became widely recognized on November 30, 2022, when OpenAI released ChatGPT, a free web-based service [4,5]. This milestone illustrated the potential of LLMs to advance toward general purpose artificial intelligence (AI) by enabling natural language–based dialogue. The continuously expanding medical knowledge has traditionally been managed through structured data storage, indexing, and query mechanisms [6] or probabilistic inference algorithms [7]. However, these conventional clinical decision support systems struggle to incorporate new information and adapt to complex clinical contexts [8]. Unlike these approaches, LLM-driven systems enable interaction in natural language, offering a more user-friendly and adaptable knowledge management tool. Such systems can be easily deployed across health care teams, including physicians and allied health care professionals. In this study, we devised a model to understand how this new LLM-based methodology could affect medical decision-making and routine clinical workflow. The process of our project and our key findings were described as follows.

## Decision-Making Aid Construction and Assessment

### Constructing an Expert System With a Free AI Tool Based on LLM

**Figure 1:**
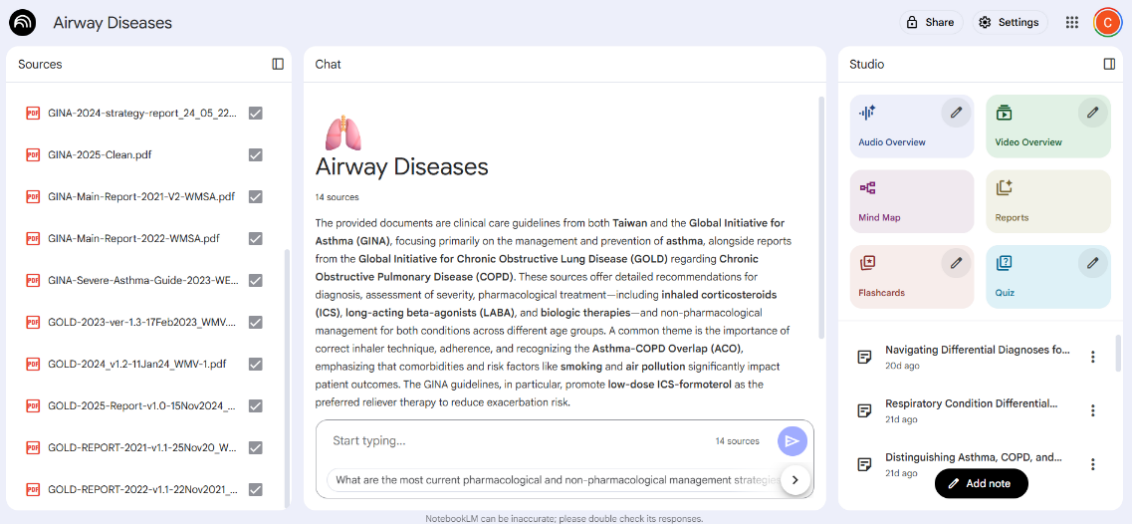
The Google NotebookLM interface.

The free AI tool Google NotebookLM, powered by the LLM Gemini 2.0 [9], provides a platform for knowledge integration through 3 components: a source input pane, a query pane, and a note-taking pane, which function collaboratively (Figure 1). We used this platform to develop a medical decision support system for airway diseases. Relevant published clinical guidelines were uploaded into the source input pane, including the GINA (Global Initiative for Asthma) guideline [10] and the GOLD (Global Initiative for Chronic Obstructive Lung Disease) guideline [11] (version 2021-2025). Additional standard documents from the Taiwan Society of Pulmonary and Critical Care Medicine [12], such as the Taiwan asthma guidelines and the pulmonary rehabilitation guidelines, were also incorporated. The responses of the system to queries were then recorded.

### Feasibility Evaluated by Pulmonologists

Query questions were selected to match the essential requirements of eligible AI tools, such as reproducibility, the ability to develop a treatment plan, and the capacity to handle speculative ideas. They were designed by a pulmonologist with expertise in airway diseases and familiarity with these guidelines, and the appropriateness (focusing on accuracy and completeness) of AI responses was judged (Likert scale 1-5) by 3 pulmonologists independently. Airway diseases were selected as the pilot scenario to evaluate the feasibility of our system because standardized clinical guidelines are widely accepted, and they involve fewer complex and controversial data points than other conditions, such as oncological diseases.

### Implementation in the Clinical Workflow

The system was introduced to physicians and allied health care professionals in the emergency department (ED), where patients with airway diseases commonly present for treatment. Feedback was collected through a questionnaire, and the responses were analyzed to assess the system’s effectiveness within the clinical workflow.

The ED setting was chosen because clinicians frequently encounter diverse conditions and require rapid access to specialist knowledge. In this context, an expert system capable of answering clinical queries has the potential to reduce the time burden on patients, frontline clinicians, and the specialists consulted.

**Figure 2:**
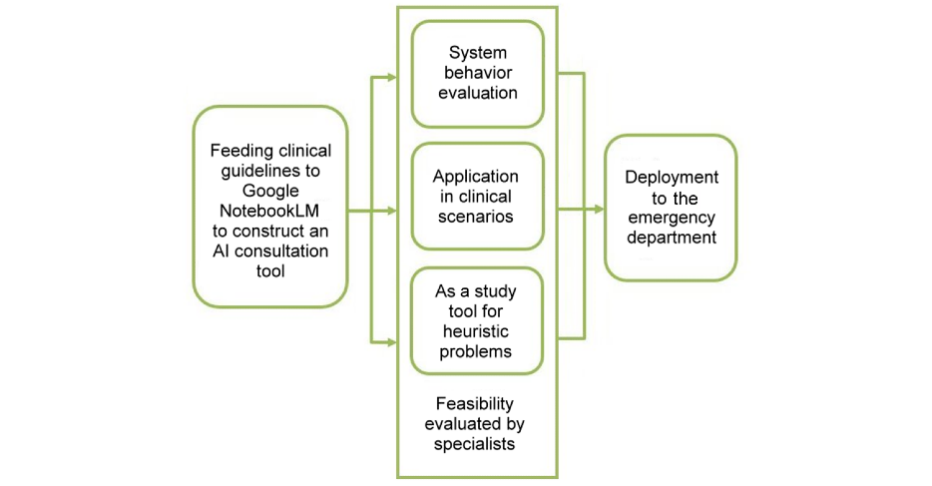
Flowchart illustrating the process of constructing our artificial intelligence (AI)–based consultation tool for medical decision-making.

The complete workflow of the project is summarized in Figure 2.

### AI Responses to Queries

#### System Behavior Evaluation

##### Test 1: Reproducibility

The system consistently generated accurate summaries and responses, each supported by appropriate and reliable references. When prompted to regenerate output from the same sources, this system produced slightly varied responses that retained identical meaning, resembling the variability typically observed in an AI chatbot interaction.

##### Test 2: Knowledge Consolidation

The asthma and chronic obstructive pulmonary disease (COPD) guidelines contained respective lists of differential diagnoses. When instructed to generate a consolidated table of differential diagnoses for airway diseases, including their distinguishing features and relevant diagnostic tests, the system successfully produced one, as shown in Table 1.

**Table 1:** Summary of the differential diagnoses for asthma, chronic obstructive pulmonary disease (COPD), and related conditions, including distinguishing symptoms, clinical features, and tests. Full version available in Multimedia Appendix 1.

Condition	Primary differential for	Differentiating symptoms and features	Differentiating tests and findings
Inducible laryngeal obstruction or vocal cord dysfunction	Both asthma and COPD	Dyspnea and inspiratory wheezing (stridor), sometimes with cough and general wheeze	Functional laryngoscopy visualizing vocal cord movement; full flow-volume curve assessing upper airway obstruction
Obesity	Both asthma and COPD	Respiratory symptoms, such as dyspnea and wheeze, that can mimic asthma or COPD, often related to deconditioning or mechanical restriction	Objective measurement of variable expiratory airflow limitation to confirm asthma in obese patients
Tracheobronchomalacia	Both asthma and COPD	Wheeze	Bronchoscopy; high-resolution chest computed tomography scan
Lung cancer	Both asthma and COPD (more common in COPD)	Breathlessness, significant weight loss, hemoptysis, night sweats, and fever	Chest radiograph or computed tomography scan; reduced diffusing capacity for carbon monoxide may be present
Alpha-1 antitrypsin deficiency	Both asthma and COPD (more commonly associated with COPD)	Shortness of breath, with a family history of early-onset emphysema	Blood test confirming alpha-1 antitrypsin deficiency
Tuberculosis	Both asthma and COPD (especially in low- and middle-income countries)	Chronic cough, hemoptysis, dyspnea and/or fatigue, fever, night sweats, anorexia, and weight loss	Sputum induction (to confirm inflammatory phenotype); high-resolution computed tomography of the chest

##### Test 3: Temporal Order Recognition

The system demonstrated the ability to recognize temporal updates. For instance, it correctly identified that the “ABCD assessment tool” for COPD was replaced by the “ABE assessment scheme” in the 2023 update of the GOLD COPD guideline [11]. When asked to provide treatment recommendations, the system appropriately referred to the most recent guideline version while disregarding outdated ones, as shown in Textbox 1.

**The chronic obstructive pulmonary disease (COPD) grouping follows the most recent version of the clinical guidelines. For full details, refer to Multimedia Appendix 2.**

**Question**

A COPD patient with mMRC=2 and CAT=11, and a history of hospitalization once in the past 1 year. What is his/her treatment suggestion?

**Answer**

...

3. GOLD Group Classification:

Combining a high symptom burden (mMRC=2, CAT=11) with a high exacerbation risk (one hospitalization), this patient is classified as GOLD Group E [GOLD 2023, p. 40, Figure 2.4; GOLD 2024, p. 29, Figure 2.5; GOLD 2025, p. 38, Figure 2.5]. This classification guides therapeutic recommendations [GOLD 2023, p. 40; GOLD 2024, p. 29; GOLD 2025, p. 38]...

##### Test 4: Memory Flush

When the COPD guidelines from 2023 onward were deselected from the source input panel, the system could still respond to questions about “ABE assessment” using information retained from previous dialogues, provided those dialogues had not been deleted using the Refresh button (Textbox 2). Interestingly, if the dialogues were deleted and no clues regarding the 2023 update were supplied, the system concluded that “ABE” might be a typographic error for “ABCD” (Textbox 3) or “creatively“ suggested that “B” referred to blood eosinophil count (Textbox 4). This indicates that the AI tool retains dialogue content until it is deliberately erased using the Refresh button.

**The system could respond using information from prior dialogues until those dialogues were removed using the Refresh button.**

**Question**

What is the ABE assessment tool?

**Answer**

The provided 2021 and 2022 reports do not contain information about merging groups C and D into a single “E” group, nor do they refer to an “ABE” assessment tool. This suggests that the change to the ABE classification, if it occurred, was introduced in a subsequent GOLD report (e.g., the GOLD 2023 report as mentioned in our previous conversation).

**A system response after a memory flush.**

**Question**

Explain the ABE assessment tool for COPD.

**Answer**

The sources provided do not contain information on an “ABE assessment tool” for COPD. However, they extensively describe the “ABCD” assessment tool used in the Global Initiative for Chronic Obstructive Lung Disease (GOLD) guidelines for COPD management. It is possible that “ABE” in your query was a typographical error for “ABCD” or refers to a concept not detailed in these documents.

**A creative response after a memory flush.**

**Question**

Explain the ABE assessment tool for COPD.

**Answer**

While the provided sources do not explicitly define or refer to an “ABE assessment tool” as a named entity for evaluating COPD, they do describe a framework for the initiation of pharmacological management that relies on assessing factors commonly associated with “A” (Symptoms), “B” (Blood Eosinophil Count), and “E” (Exacerbation Risk).

##### Test 5: Handling Multiple Languages

Leveraging Google’s multilingual capability, the system could accept a query in Chinese and respond in either English or Chinese. When asked to compare the 2022 Taiwan adult asthma clinical care guidelines, written entirely in Chinese, with the GINA guideline, the system managed to perform the task without difficulty. Language switching did not appear to affect system performance.

#### Application in Clinical Scenarios

##### Scenario 1: Requesting Patient Management Suggestions Based on a Simulated Medical Record

As illustrated in Textbox 5, the system generated guideline-based recommendations that were clinically useful.

**Helpful recommendations generated by the system, supported by guideline-based reasoning. For a complete explanation, refer to Multimedia Appendix 3.**

**Question**

I have a patient diagnosed of COPD adequately maintained with Pulmicort, 2 puffs bid. However, he suffered from more and more dyspnea while walking. What is the management suggestion?

**Answer**

Based on the information in the sources, here are some management suggestions for your patient with COPD who is experiencing increasing dyspnea on walking despite being maintained on Pulmicort (an inhaled corticosteroid - ICS):

1. Consider adding a long-acting bronchodilator...
2. Consider a combination inhaler containing ICS and LABA...
3. Optimize inhaled therapy. Ensure that the patient...
4. Consider pulmonary rehabilitation...
5. Reassess lung function and consider other diagnoses...
6. Review for modifiable risk factors...
7. Consider stepping up ICS dose (with caution)...
8. Assess for features of asthma-COPD overlap (ACO)...
9. Follow-up and reassess...

##### Scenario 2: Logical Question

The system addressed a logical question regarding the unavailability of pulmonary function testing by providing a structured, entry-by-entry response. It explained the implications separately for COPD and asthma, as shown in Textbox 6.

**The system presented a comparative response for chronic obstructive pulmonary disease (COPD) and asthma. For the complete answer, refer to Multimedia Appendix 4.**

**Question**

Is pulmonary function test necessary for diagnosis? What if there is no pulmonary function test accessible?

**Answer**

Here’s how pulmonary function tests relate to diagnosing airway diseases, and what to do if these tests aren’t accessible:

- COPD Diagnosis: Spirometry is essential to confirm a COPD diagnosis...
- Asthma Diagnosis: Asthma diagnosis relies on a combination of characteristic symptom patterns and evidence of variable expiratory airflow limitation...
- Role of Lung Function Testing: Lung function testing is essential to confirm...
- Spirometry: Forced spirometry is the most reproducible and objective measurement of airflow obstruction. It measures...
- When Spirometry Isn’t Available for Asthma Diagnosis:
  Peak expiratory flow (PEF) can be used, although it is less reliable...
  Assess the patient for a characteristic pattern of respiratory symptoms.
  Consider a therapeutic trial with SABA and low-dose ICS...
  Confirm variable expiratory airflow limitation by PEF. A ≥20% improvement in PEF...
  Improvement in symptoms and PEF after a 4-week therapeutic trial...
- When Spirometry Isn’t Available for COPD Diagnosis: When routine spirometry is not available, home measurement of peak expiratory flow (PEF) combined with validated patient questionnaires could be used to support or refute a possible diagnosis of COPD...

##### Scenario 3: Personalized Rehabilitation Planning

The system generated a tailored rehabilitation plan and schedule that took into account an advanced age condition, as shown in Textbox 7.

**The system produced a detailed, individualized rehabilitation plan for an extremely older adult patient with chronic obstructive pulmonary disease (COPD) and ischemic heart disease. The complete plan is available in Multimedia Appendix 5.**

**Question**

I have a female patient, age 95, with COPD and ischemic heart disease. Please make a rehabilitation plan and schedule for her.

**Answer**

For your 95-year-old female patient with COPD and ischemic heart disease, a highly individualized, comprehensive, and carefully supervised rehabilitation plan is crucial, taking into accounts her advanced age and multiple comorbidities. This plan draws primarily from the Taiwan Pulmonary Rehabilitation Practical Guideline and the Global Initiative for Chronic Obstructive Lung Disease (GOLD) reports.

I. Overall Approach and Guiding Principles

1. Personalized and Integrated Care:...
2. Specialist Supervision:...
3. Holistic Goals:...

II. Initial Comprehensive Assessment

- Patient Goals and Preferences:...
- Medical History and Physical Examination:...

...

III. Rehabilitation Setting and Schedule

1. Setting: For a 95-year-old patient with significant comorbidities, a supervised setting is highly recommended, such as...
2. Duration: Optimal benefits from pulmonary rehabilitation programs are typically achieved within 6 to 8 weeks...
3. Frequency: Supervised exercise training at least twice weekly is recommended.

IV. Components of the Rehabilitation Plan

1. Exercise Training:...
2. Education and Self-Management:...

...

V. Follow-up and Monitoring

- Regular Review:...
- Response Assessment:...

...

##### Scenario 4: Vague Question

In this case, the system transformed the ambiguous concept of “prospect” into practical categories of “future challenges” and “research directions.” This approach provided a meaningful response, as illustrated in Textbox 8.

**The system interpreted the vague query as a request for practical insights. The complete answer is provided in Multimedia Appendix 6.**

**Question**

What is the future prospect of airway diseases and their treatment?

**Answer**

The sources discuss future challenges and research directions for airway diseases such as asthma and Chronic Obstructive Pulmonary Disease (COPD).

Asthma:

- Despite improvements in asthma care and effective medications, many patients worldwide do not benefit from these advances due to inadequate medical facilities and financial resources.
- Future research should focus on clinical and physiological characteristics, biomarkers, outcomes, and underlying mechanisms in patients with respiratory symptoms or chronic airflow limitation...

COPD:

- More research is needed to guide better recognition and safe, effective treatment of COPD.
- There’s a need to study clinical and physiological characteristics, biomarkers, outcomes and underlying mechanisms in broad populations of patients with respiratory symptoms or with chronic airflow limitation.
- Future studies should focus on defining phenotypes and mechanisms to ensure the safety of pharmacological treatments...

##### Scenario 5: Out-of-Scope Questions

The system appropriately declined to answer questions that were beyond the scope of the available sources. It did not fabricate factitious responses, unlike many chatbots.

#### As a Study Tool for Heuristic Problems

##### Problem 1: Version Comparison and Trend Analysis

When asked to compare guideline versions across publication years, the system clearly outlined the differences between them. Additionally, when requested to analyze trends in pharmacological use for airway diseases over the past 5 years, it generated a comprehensive summary, as shown in Textbox 9.

**The system analyzed the trend in pharmacological use for airway diseases across the years. A complete list is available in Multimedia Appendix 7.**

**Question**

Find the trend in pharmacological use for COPD and asthma in the past 5 years.

**Answer**

In the past five years, pharmacological treatment for both Chronic Obstructive Pulmonary Disease (COPD) and asthma has seen a clear trend towards more individualized, exacerbation-focused, and strategically combined therapies, with a strong emphasis on proper inhaler use and minimizing oral corticosteroid exposure.

Overarching Trends for Both COPD and Asthma (Past 5 Years)

1. Individualized and Patient-Centered Care:...
2. Focus on Exacerbation Reduction:...
3. Emphasis on Inhaler Technique and Adherence:...
4. Minimizing Oral Corticosteroid (OCS) Use:...

Trends in COPD Pharmacological Treatment (Past 5 Years): The GOLD reports (2021, 2022, 2023, 2024, 2025) and the 2023 Taiwan COPD Clinical Care Guidelines emphasize these trends:

1. Long-Acting Bronchodilators (LABA and/or LAMA) as First-Line:...
2. Strategic Use of Inhaled Corticosteroids (ICS):...
3. Increasing Role of Triple Therapy (LABA+LAMA+ICS):...

...

Trends in Asthma Pharmacological Treatment (Past 5 Years): GINA reports (2021, 2022, 2023, 2024, 2025) and the 2022 Taiwan Adult Asthma Clinical Care Guidelines highlight significant shifts:

1. Shift Away from SABA Monotherapy:...
2. Introduction of Two Treatment Tracks (GINA):...
3. ICS as the Cornerstone of Controller Therapy:...

...

##### Problem 2: Further Reading Suggestions

When asked to recommend additional readings on intractable asthma, the system searched both the source text and the reference lists it contained. However, it did not suggest any external articles or books. Notably, the system interpreted the term “intractable asthma” as both “severe asthma” and “difficult-to-treat asthma” to ensure comprehensive coverage, as shown in Textbox 10.

**Suggested further reading items provided by the system. For the complete list, refer to Multimedia Appendix 8.**

**Question**

Can you suggest any further readings about the treatment for intractable asthma?

**Answer**

For further readings about the treatment of intractable asthma, which is often referred to in the sources as severe asthma or difficult-to-treat asthma, you may find the following publications insightful. These sources provide detailed information on diagnosis, management, and treatment options for patients whose asthma remains uncontrolled despite standard therapies:

Comprehensive Guidelines and Reviews:

- Global Strategy for Asthma Management and Prevention (GINA): The GINA reports are frequently referenced for their comprehensive guidance on asthma management. Specifically, the sections within these reports on “Difficult-to-treat and severe asthma” provide a roadmap for assessment and management. You can refer to the detailed sections in the provided GINA reports, such as:
  GINA-2023-Report:...
  GINA-2024-strategy-report:...
  GINA-2025-Clean:...

...Pharmacological Interventions:

- Biologic Therapies:
  EAACI Biologicals Guidelines-Recommendations for severe asthma: Agache I et al. offer guidelines and recommendations for biologics in severe asthma.
  Tezepelumab:...

...

- Add-on treatments (nonbiologic):
  Tiotropium:...
  Azithromycin:...
  Oral Corticosteroids (OCS):...

Diagnostic and Management Strategies:

- Sputum-guided treatment...
- Bronchial Thermoplasty:...
- Importance of Adherence and Inhaler Technique:...

These readings should provide a comprehensive overview of the current understanding and treatment approaches for intractable asthma.

##### Problem 3: Speculative Question

We designed a question regarding the interchangeability of questionnaires used for evaluating COPD and asthma. Our system provided a reasonable response, as shown in Textbox 11.

**The system addressed this speculative question and provided a supporting rationale.**

**Question**

Can we evaluate asthmatic patients with questionnaires designed for COPD? Can we evaluate COPD patients with questionnaires designed for asthma?

**Answer**

Asthma and COPD can be difficult to distinguish, especially in older patients and smokers, and these conditions may overlap. Some patients are assigned both diagnoses. While some questionnaires might capture general respiratory symptoms that could be present in both asthma and COPD, it is best to use disease-specific questionnaires to ensure the most appropriate and accurate assessment. There is no evidence that asthma questionnaires can be used for COPD patients, and vice versa.

##### Problem 4: Diagnostic Skill Training Tool

We instructed the system to generate a simulated case with symptoms and pulmonary function values to test our diagnostic skills. During this exercise, we compared our reasoning and diagnosis with the system’s answer to strengthen diagnostic ability, as shown in Textbox 12.

**The system generated a simulated case to facilitate diagnostic training. For the answer, diagnostic rationale, and treatment recommendation, refer to Multimedia Appendix 9.**

**Question**

Please make a case with symptoms and pulmonary function values to test our diagnostic skills.

**Answer**

Simulated Patient: Mr David Lee

Patient Background: Mr David Lee is a 58-year-old male with a 25 pack-year smoking history, though he states he is currently trying to quit. He reports a chronic cough with some mucus production for the past 8 years. He also experiences intermittent shortness of breath on exertion, particularly when climbing stairs. Lately, he’s noticed that his breathing feels tighter in the mornings, and he occasionally hears a whistling sound (wheeze) when breathing out, especially after exposure to cold air. These wheezing episodes are variable in intensity, and he notes that his symptoms sometimes improve with a relative’s “asthma inhaler” (a short-acting beta2-agonist, SABA).

Symptoms:

- Chronic cough with mucus production (8 years duration).
- Intermittent dyspnea on exertion (eg, climbing stairs).
- Morning chest tightness.
- Occasional expiratory wheezing, variable in intensity, exacerbated by cold air.
- Reports temporary improvement with SABA.
- Significant smoking history (25 pack-years).

Pulmonary Function Values:

- Pre-Bronchodilator Spirometry:
  Forced Expiratory Volume in 1 second (FEV1): 60% of predicted
  Forced Vital Capacity: 80% of predicted
  FEV1/Forced Vital Capacity Ratio: 65% (indicating airflow limitation)
- Post-Bronchodilator Spirometry (after administering 400 mcg of salbutamol, a SABA):...

-----------------------------------------------------------------------------------

Diagnosis, Reasons, and Treatment Suggestion for Mr David Lee

...

#### Pulmonologists’ Judgment on AI Responses to Queries

Most of the 3 pulmonologists’ ratings (56/84, 67%) regarding AI responses were above average (Likert scale 4-5), and only 5% (4/84) were below average (Likert scale 1-2). The interrater reliability was moderate for accuracy (intraclass correlation coefficient=0.612; *P*<.001) and good for completeness (intraclass correlation coefficient=0.773; *P*<.001) based on a 2-way random-effects model with absolute agreement and single measures options [13]. We also noted that AI responses received more (187/333, 56%) credits from the raters for query questions that required more intelligence (adaptation, deduction, analysis, and planning) to answer, such as scenarios 1 to 3 and problems 1 to 4, than for the other questions. This finding further supports the feasibility of our system.

#### Responses From the Field Tests in the ED

We collected responses from 20 participants, including 3 physicians, 3 nurse practitioners, 11 ED nurses, and 3 respiratory therapists. Among them, 80% (16/20) expressed interest in the system (Kolmogorov-Smirnov [K-S] *D*=0.362; *P*=.008; indicating a deviation from normality). All these participants had prior experience consulting a chatbot. In total, 60% (12/20) of the participants were aware of and ever disturbed by the issue of AI hallucination (K-S *D*=0.238; *P*=.18; indicating a normal distribution). All participants in this group thought that guideline-based reasoning provided by the system was essential in clinical practice.

In total, 55% (11/20) of the participants believed that the system could reduce specialty consultation time (K-S *D*=0.223; *P*=.24; indicating a normal distribution). Importantly, all participants indicated that the system could enhance their knowledge and diagnostic ability in airway diseases or increase their confidence in explaining conditions to patients.

## Discussion

### Statistical Findings

Previous studies on LLM-based medical decision support have reported various outcomes [14,15]. These discrepancies may reflect differences in participants’ expertise, the difficulty of test questions, and the dimensions measured in study designs. In our evaluation of consultation time, the K-S test indicated a normal distribution of responses. When analyzing only physicians’ responses, we observed a trend toward favorable outcomes, although this did not reach statistical significance (*P*=.24) across all participants. Thus, the system shows potential for reducing physicians’ consultation time, but larger and more comprehensive studies are required to confirm its effectiveness.

### What We Expect of a Medical AI Consultation Tool

#### AI Hallucination and AI Ethics in Medicine

Since the widespread adoption of LLM-based chatbots, users have become increasingly concerned about AI hallucination, that is, plausible but unfounded responses generated without a factual basis. Such errors are unacceptable in medical practice. Amiot and Potier [16] recently emphasized that hallucination is particularly difficult to detect in a health care context. Fortunately, the design of the Google NotebookLM platform mitigates this risk by providing answers supported by explicit references to authoritative sources. With such verifiable citations, concerns about errors, biases, ethics, and safety from autonomous AI actions are substantially reduced.

#### Beyond a Decision-Making Aid

The project aims to provide a clinical decision-making aid for the diagnosis and management of airway diseases and to assess how this AI tool affected medical staff in the ED during the consultation process. As Wang et al [17] noted, an AI system could uncover subtle relationships in medical data that may elude both traditional decision support tools and human experts. During system development, pulmonologists found that the tool not only consolidated their expertise but also offered novel insights and opportunities to explore speculative ideas, as described in test problems 1 and 3. For medical trainees, the system functions as an effective educational tool, providing not only suggestions but also the underlying reasons.

### Limitations Inherent to LLMs

#### Scope of External Knowledge Considered

NotebookLM generates content strictly from user-provided sources. Because general “common sense” information is often absent from “specialized” clinical guidelines, excluding external knowledge can considerably limit the scope of the system’s responses. Moreover, the extent to which external information is incorporated into outputs is undisclosed and therefore unpredictable.

#### Inability to Pose Differentiating Questions

LLM-based systems generate responses in the form of summaries or abstracts but do not query users. However, accurate clinical decision-making often requires additional patient-specific data obtained through differentiating questions. For example, clarifying whether a patient is a smoker or a nonsmoker or identifying ethnic background (Asian or White) may guide diagnostic pathways. If the system could formulate such discriminative questions and allow users to provide input—ideally guided by Bayesian theorem [18]—its clinical utility would be markedly enhanced. We did not observe this feature in the current implementation.

### Future Directions

Once the pilot project is fully operational, the expert system can be refined along several pathways. First, the system should adapt its responses to the user’s professional role. For example, when handling a consultation request, it should provide only essential surgical information to an internist while delivering more comprehensive surgical details to a surgeon.

Second, although our study focused on airway diseases and the system demonstrated generally positive performance, its generalizability to other specialties remains uncertain. Disciplines such as oncology, where problems are more complex and controversies are common, may present challenges. Further research is required to determine whether the model can provide reliable and clinically meaningful output in these domains.

Finally, because decision-making, studying, and education are central components of our medical practice routines, integrating such a system into the existing health care information systems presents a logical next step. We expect that this integration could streamline clinical workflows and improve automation across multiple aspects of care delivery.

### Conclusions

Our findings suggest that this approach is highly customizable, cost efficient, and accessible to clinicians and allied health care professionals without any computer coding experience in managing airway diseases. It provides convincing guideline-based recommendations, increases the staff’s medical literacy, and potentially saves physicians’ time spent on consultation. As AI tools gain widespread adoption, we believe that, if validated across additional medical specialties, this methodology could establish a new paradigm in clinical practice. While acknowledging the potential of AI, we emphasize the continued need for human judgment and oversight in evaluating and applying AI-generated recommendations.

## Appendix

Multimedia Appendix 1 Unabridged version of Table 1.

Multimedia Appendix 2 Unabridged version of Textbox 1.

Multimedia Appendix 3 Unabridged version of Textbox 5.

Multimedia Appendix 4 Unabridged version of Textbox 6.

Multimedia Appendix 5 Unabridged version of Textbox 7.

Multimedia Appendix 6 Unabridged version of Textbox 8.

Multimedia Appendix 7 Unabridged version of Textbox 9.

Multimedia Appendix 8 Unabridged version of Textbox 10.

Multimedia Appendix 9 Unabridged version of Textbox 12.

## Abbreviations

AI: artificial intelligence
COPD: chronic obstructive pulmonary disease
ED: emergency department
GINA: Global Initiative for Asthma
GOLD: Global Initiative for Chronic Obstructive Lung Disease
K-S: Kolmogorov-Smirnov
LLM: large language model
