# Supplementary material for: Improving Clinical Decision-Making in Treating Airway Diseases With an Expert System Built Upon the Free AI Tool Google NotebookLM
Source: JMIR Med Inform. 2026 Jan 29;14:e78567. doi: 10.2196/78567 (PMC12902755; doi:10.2196/78567)
Supplement: Multimedia Appendix 1 [file medinform_v14i1e78567_app1.docx]

Appendix 1. Unabridged version of Table 1.

Here is a table summarizing the differential diagnoses for asthma, Chronic Obstructive Pulmonary Disease (COPD), and other conditions with similar symptoms, highlighting their differentiating features and tests:

**Differential Diagnoses of Asthma, COPD, and Other Conditions with Similar Symptoms**

| **Condition** | **Primary Differential for** | **Differentiating Symptoms/Clinical Features** | **Differentiating Tests/Findings** |
| --- | --- | --- | --- |
| **Asthma vs. COPD: Key Differentiators** |  |  |  |
| **Asthma** | COPD | **Symptoms are variable** over time and intensity, often **worse at night or early morning**, and triggered by exercise, allergens, cold air, or viral infections. Often commences in childhood, with a past or family history of allergic disease (e.g., eczema, allergic rhinitis). | **Spirometry:** Characterized by **variable expiratory airflow limitation**. Often shows **marked bronchodilator responsiveness** (increase in FEV1 of ≥12% and ≥200 mL). **DLCO** (diffusing capacity of the lungs for carbon monoxide) is typically normal or slightly elevated. **Imaging (HRCT)** is usually normal, but may show air trapping or increased bronchial wall thickness. **Biomarkers:** Elevated **FeNO** (>50 ppb in adults/adolescents; >35 ppb in children) or **blood eosinophils** (above national/regional reference range) can support Type 2 asthma diagnosis, but are not exclusive to asthma. |
| **Chronic Obstructive Pulmonary Disease (COPD)** | Asthma | **Symptoms are persistent** and often progressive, including dyspnea, chronic cough, and sputum production. Typically develops in adulthood, with a strong history of smoking or significant environmental exposures. Chronic cough is often the first symptom and may be discounted by the patient as a consequence of smoking. | **Spirometry:** Confirmed by **persistent, non-fully reversible airflow obstruction** (post-bronchodilator FEV1/FVC < 0.7). **DLCO** is often reduced. **Imaging (HRCT)** frequently reveals emphysema, gas trapping, bronchial wall thickening, and features of pulmonary hypertension. **Biomarkers:** Eosinophilia may be present in COPD, including during exacerbations. High blood eosinophil counts (≥300 cells/µL) can guide corticosteroid use. FeNO levels are generally lower in current smokers. |
| **Asthma-COPD Overlap (ACO) / Asthma+COPD** | Both Asthma & COPD | Patients have **persistent airflow limitation combined with clinical features of both asthma and COPD**. These patients generally experience a greater burden of symptoms, more frequent exacerbations, poorer quality of life, and higher mortality compared to those with either condition alone. | **Spirometry:** Shows persistent airflow limitation (FEV1/FVC < 0.7) but may also exhibit significant bronchodilator reversibility. **DLCO** is often reduced. |
| **Other Respiratory Conditions (Primarily Differentiated from Asthma)** |  |  |  |
| **Chronic Upper Airway Cough Syndrome (UACS) / Postnasal Drip** | Asthma | Sneezing, itching, blocked nose, throat-clearing, and a persistent non-productive cough. | No specific lung function abnormalities directly related to asthma. |
| **Cough Variant Asthma (CVA)** | Asthma | Persistent cough as the principal or only symptom, often worse at night or with exercise, and typically non-productive (though it can be productive). Wheezing and bronchodilator responsiveness may develop later. | **Bronchial provocation test:** Airway hyperresponsiveness (AHR) when spirometry is otherwise normal. Many patients have sputum eosinophilia and may have elevated FeNO. |
| **Non-asthmatic Eosinophilic Bronchitis (NAEB)** | Asthma | Cough and sputum eosinophilia. | **Spirometry and airway responsiveness:** Both are normal. |
| **Inhaled Foreign Body** | Asthma | Sudden onset of symptoms and often unilateral wheeze. | - |
| **Bronchopulmonary Dysplasia (BPD)** | Asthma (children) | History of pre-term birth and symptoms since birth. | - |
| **Cystic Fibrosis (CF)** | Asthma | Excessive cough and mucus production, often with gastrointestinal symptoms and recurrent infections. | - |
| **Chronic Rhinosinusitis (with/without nasal polyps)** | Asthma | Symptoms like sneezing, itching, blocked nose, and throat-clearing. | **CT of the sinuses:** Can identify changes indicative of chronic rhinosinusitis. |
| **Post-infectious cough** | Asthma | An isolated non-productive cough that persists after a respiratory infection. | - |
| **ACE inhibitor-induced cough** | Asthma | An isolated non-productive cough in a patient taking an ACE inhibitor. | Cough resolves upon discontinuation of the ACE inhibitor. |
| **Aspirin-exacerbated respiratory disease (AERD)** | Asthma | (Mentioned as an airway condition to investigate for in specialist care). | - |
| **Respiratory Conditions (Primarily Differentiated from COPD)** |  |  |  |
| **Pneumonia** | COPD (exacerbations) | Worsening respiratory symptoms (dyspnea, cough, sputum), often accompanied by fever. | **Chest X-ray:** Detects infiltrates or consolidation indicative of pneumonia. |
| **Other Conditions with Overlapping or Similar Symptoms (Can be Confused with Asthma or COPD)** |  |  |  |
| **Congestive Heart Failure / Cardiovascular Disease** | Both Asthma & COPD | Wheezing, breathlessness, and cough, often worse with exercise or at night, particularly in older adults. May present with cardiac murmurs. | **ECG, Chest X-ray, Plasma BNP, Echocardiography:** Used to assess cardiac function. **DLCO** is often reduced in cardiac disease. |
| **Inducible Laryngeal Obstruction (ILO) / Vocal Cord Dysfunction (VCD)** | Both Asthma & COPD | Dyspnea and inspiratory wheezing (stridor), sometimes with cough and general wheeze. | **Functional laryngoscopy:** Visualizes vocal cord movement. **Full flow-volume curve:** Can assess for upper airway obstruction. |
| **Obesity** | Both Asthma & COPD | Respiratory symptoms like dyspnea and wheeze that can mimic asthma or COPD, often related to deconditioning or mechanical restriction. | **Objective measurement of variable expiratory airflow limitation** is important to confirm asthma in obese patients. |
| **Tracheobronchomalacia** | Both Asthma & COPD | Wheeze. | **Bronchoscopy, high-resolution chest CT**. |
| **Lung Cancer** | Both Asthma & COPD (more common in COPD) | Breathlessness, significant weight loss, hemoptysis, night sweats, and fever. | **Chest X-ray or CT scan**. Reduced DLCO may be present. |
| **Alpha-1 Antitrypsin Deficiency (AATD)** | Both Asthma & COPD (more associated with COPD) | Shortness of breath, with a family history of early emphysema. | **Blood test:** Confirms AATD. |
| **Tuberculosis (TB)** | Both Asthma & COPD (especially in LMICs) | Chronic cough, hemoptysis, dyspnea, and/or fatigue, fever, night sweats, anorexia, weight loss. | **Sputum induction:** Can confirm inflammatory phenotype. **HRCT**. |
| **HIV/AIDS-associated lung diseases** | Both Asthma & COPD (LMICs) | Symptoms similar to TB, prominent in low- and middle-income countries. | - |
| **Parasitic or fungal lung diseases** | Both Asthma & COPD (LMICs) | Often linked to Type 2 inflammation and eosinophilia, prevalent in low- and middle-income countries. | **Fungal precipitins** (including Aspergillus). **Parasite testing** (e.g., Strongyloides serology, stool examination) if blood eosinophils ≥300/µL. |
| **Pulmonary Embolism** | COPD (exacerbations) | Worsening dyspnea and other respiratory symptoms. | **D-dimer test**. |
| **COVID-19 Infection** | COPD | Cough, breathlessness, often accompanied by fever (>60%), fatigue, confusion, diarrhea, nausea, vomiting, muscle aches, anosmia, dysgeusia, and headaches. Rapid deterioration in lung function can occur. | **RT-PCR testing** for SARS-CoV-2. Common findings include lymphopenia, elevated D-dimer, C-reactive peptide (CRP), procalcitonin, creatinine kinase, transaminases, creatinine, and lactate dehydrogenase (LDH). |
| **Depression and Anxiety** | Both Asthma & COPD | Fatigue, reduced physical activity, and can significantly contribute to symptom reporting and poor quality of life, making it difficult to distinguish from asthma or COPD symptoms. | **Mental health assessment** and disease-specific psychiatric diagnostic tools. |
| **Hyperventilation, Dysfunctional Breathing** | Both Asthma & COPD | Dizziness, light-headedness, peripheral tingling (paresthesia), and sighing. | - |
| **Primary Ciliary Dyskinesia (PCD)** | Both Asthma & COPD (especially children) | Recurrent infections, productive cough, and sinusitis. | - |
| **Congenital Heart Disease** | Both Asthma & COPD (especially children) | Presence of cardiac murmurs. | **Echocardiography, chest X-ray**. |
| **Bronchiectasis** | Both Asthma & COPD | Recurrent infections, productive cough, and excessive mucus production. | **High-resolution CT (HRCT) of the lungs:** Clearly identifies bronchiectasis. |
| **Chronic Bronchitis (without spirometric obstruction)** | COPD | Chronic cough with sputum production (mucous, mucopurulent, or purulent), often linked to smoking. Can occur in patients without airflow obstruction. | **Spirometry:** Post-bronchodilator FEV1/FVC is normal (i.e., not meeting COPD criteria). |
| **Obstructive Sleep Apnea (OSA)** | Both Asthma & COPD | Can contribute to respiratory symptoms (dyspnea, poor sleep quality), and poor quality of life, sometimes exacerbating asthma control. | **Sleep study:** Confirms diagnosis of OSA. |
