# Supplementary material for: Improving Clinical Decision-Making in Treating Airway Diseases With an Expert System Built Upon the Free AI Tool Google NotebookLM
Source: JMIR Med Inform. 2026 Jan 29;14:e78567. doi: 10.2196/78567 (PMC12902755; doi:10.2196/78567)
Supplement: Multimedia Appendix 2 [file medinform_v14i1e78567_app2.docx]

Appendix 2. Unabridged version of Textbox 1

For a COPD patient with **mMRC=2**, **CAT=11**, and a history of **hospitalization once in the past year**, the treatment suggestion is based on classifying their Global Initiative for Chronic Obstructive Lung Disease (GOLD) group according to their symptom burden and exacerbation risk.

Here's the assessment and recommended treatment:

1. **Symptom Burden Assessment:**

    ◦ The **Modified British Medical Research Council (mMRC) Questionnaire** assesses breathlessness. An mMRC score of **<2** indicates less breathlessness, while **≥2** indicates more breathlessness [GOLD 2023, p. 38; GOLD 2024, p. 29; GOLD 2025, p. 38]. With an mMRC score of **2**, this patient falls into the **"high symptom burden"** category.

    ◦ The **COPD Assessment Test (CAT™)** assesses overall symptoms. A CAT score of **<10** indicates fewer symptoms, while **≥10** indicates more symptoms [GOLD 2023, p. 38; GOLD 2024, p. 29; GOLD 2025, p. 38]. With a CAT score of **11**, this patient also falls into the **"high symptom burden"** category.

    ◦ Since both measures indicate a high symptom burden, the patient is categorized as having **high symptoms**.

2. **Exacerbation Risk Assessment:**

    ◦ Exacerbation risk is determined by the history of moderate and severe exacerbations in the past year.

        ▪ **Low risk:** 0 or 1 moderate exacerbation in the past year, with no hospitalizations [GOLD 2023, p. 39; GOLD 2024, p. 29; GOLD 2025, p. 38].

        ▪ **High risk:** ≥2 moderate exacerbations OR **≥1 hospitalization** in the past year [GOLD 2023, p. 39; GOLD 2024, p. 29; GOLD 2025, p. 38].

    ◦ Given the history of **one hospitalization in the past year**, the patient is in the **"high exacerbation risk"** category.

3. **GOLD Group Classification:**

    ◦ Combining a **high symptom burden** (mMRC=2, CAT=11) with a **high exacerbation risk** (one hospitalization), this patient is classified as **GOLD Group E** [GOLD 2023, p. 40, Figure 2.4; GOLD 2024, p. 29, Figure 2.5; GOLD 2025, p. 38, Figure 2.5]. This classification guides therapeutic recommendations [GOLD 2023, p. 40; GOLD 2024, p. 29; GOLD 2025, p. 38].

4. **Treatment Suggestions for GOLD Group E:**

    ◦ **Initial Pharmacological Management:**

        ▪ The recommended initial pharmacological treatment for GOLD Group E is a **combination of a Long-Acting Muscarinic Antagonist (LAMA) and a Long-Acting Beta2-Agonist (LABA)** [GOLD 2024, p. 49, Figure 3.7; GOLD 2025, p. 55, Figure 3.7]. This dual bronchodilator therapy aims to reduce symptoms and the risk of future exacerbations [GOLD 2024, p. 47; GOLD 2025, p. 54].

        ▪ **Inhaled Corticosteroids (ICS)** may be added if the patient has a **blood eosinophil count ≥300 cells/µL** [GOLD 2024, p. 49, Figure 3.7; GOLD 2025, p. 55, Figure 3.7]. ICS are also strongly favored for patients with a **history of asthma or concomitant asthma** [GINA 2024, p. 81; GINA 2025, p. 99]. It is important to **avoid high-dose ICS** due to the associated risk of pneumonia [GINA 2024, p. 78, 81; GINA 2025, p. 93, 96]. ICS should not be used as monotherapy without LABA and/or LAMA [GINA 2023, p. 69, 72; GINA 2024, p. 81; GINA 2025, p. 96, 99].

        ▪ All patients should be prescribed **rescue short-acting bronchodilators (SABAs)** for immediate symptom relief [GOLD 2024, p. 49; GOLD 2025, p. 55, 383].

    ◦ **Non-Pharmacological Management:**

        ▪ Patients with a high symptom burden and risk of exacerbations (including Group E) should be strongly encouraged to participate in a **formal pulmonary rehabilitation program** [GOLD 2024, p. 57, 289; GOLD 2025, p. 64, 389]. This program should include setting patient goals and be structured to consider individual COPD characteristics and comorbidities [GOLD 2024, p. 57; GOLD 2025, p. 389]. Pulmonary rehabilitation has been shown to improve exercise capacity, symptoms, and quality of life across all grades of COPD severity [GOLD 2023, p. 185; GOLD 2024, p. 281; GOLD 2025, p. 377, 404].

        ▪ **Education and self-management coaching** are crucial for patients to manage their COPD daily [GOLD 2024, p. 57; GOLD 2025, p. 64]. Providing a **written action plan** is part of this guidance [GOLD 2024, p. 57, 442; GOLD 2025, p. 64, 381].

        ▪ **Smoking cessation** advice and resources should be provided if the patient is a smoker [GOLD 2024, p. 57, 68; GOLD 2025, p. 52, 64, 78].

        ▪ Appropriate **vaccinations** (e.g., influenza, pneumococcal, COVID-19, Tdap, Zoster) are recommended for all COPD patients [GOLD 2024, p. 57, 63, 127; GOLD 2025, p. 52, 64, 76, 380].

        ▪ **Comorbidities** should be actively sought and treated appropriately, as they can influence health status, hospitalizations, and mortality independently of COPD severity [GOLD 2024, p. 29-30, 57; GOLD 2025, p. 38, 41, 64, 371].
