# Supplementary material for: Improving Clinical Decision-Making in Treating Airway Diseases With an Expert System Built Upon the Free AI Tool Google NotebookLM
Source: JMIR Med Inform. 2026 Jan 29;14:e78567. doi: 10.2196/78567 (PMC12902755; doi:10.2196/78567)
Supplement: Multimedia Appendix 3 [file medinform_v14i1e78567_app3.docx]

Appendix 3. Unabridged version of Textbox 5.

Based on the information in the sources, here are some management suggestions for your patient with COPD who is experiencing increasing dyspnea on walking despite being maintained on Pulmicort (an inhaled corticosteroid - ICS):

1. **Consider adding a long-acting bronchodilator.** For patients with persistent breathlessness or exercise limitation on bronchodilator monotherapy, the use of two long-acting bronchodilators is recommended. Since your patient is currently on ICS monotherapy, adding a long-acting beta2-agonist (LABA) or a long-acting muscarinic antagonist (LAMA) could help alleviate his dyspnea.

- The 2023 GINA report suggests that for asthma, pharmacotherapy is based on ICS to reduce the risk of severe exacerbations and death and to improve symptom control, with add-on treatment as required, e.g., add-on LABA and/or LAMA. While this is for asthma, the principle of adding bronchodilators for symptom control alongside ICS might be relevant to consider in COPD as well.
- The GOLD reports indicate that pharmacotherapy for stable COPD starts with symptomatic treatment with long-acting bronchodilators (LABA and LAMA).

2. **Consider a combination inhaler containing ICS and LABA.** The GINA 2023 report mentions moderate- to high-dose ICS-LABA maintenance treatment. Switching your patient to a combination inhaler containing both Pulmicort (or another ICS) and a LABA might be a convenient way to provide both anti-inflammatory and bronchodilator effects.

3. **Optimize inhaled therapy.** Ensure that the patient is using their Pulmicort inhaler with correct technique and is adherent to the prescribed twice-daily regimen. Incorrect inhaler technique can significantly reduce the effectiveness of the medication.

4. **Consider pulmonary rehabilitation.** Pulmonary rehabilitation, which includes exercise training and education, improves exercise capacity, symptoms (like dyspnea), and quality of life across all grades of COPD severity. Referral to a pulmonary rehabilitation program could significantly benefit your patient's dyspnea on exertion.

5. **Reassess lung function and consider other diagnoses.** The GINA 2023 report suggests that if a patient with variable respiratory symptoms but no variable airflow limitation has normal spirometry (FEV1 > 70% predicted), considering stepping down ICS and reassessing, then considering a bronchial provocation test or repeating bronchodilator responsiveness is appropriate. However, since your patient has diagnosed COPD, this might be less relevant.

- If spirometry was previously normal, the GINA 2023 report also suggests considering other diagnoses if FEV1 is still normal. While COPD is diagnosed based on post-bronchodilator FEV1/FVC < 0.7, a reassessment of lung function might be useful to understand the current degree of airflow limitation.
- Consider other causes of dyspnea, especially if the symptoms are disproportionate to the degree of airflow obstruction. The sources list several conditions that can cause dyspnea, such as cardiac failure, pulmonary embolism, parenchymal lung disease, central airway obstruction, and inducible laryngeal obstruction. Comorbidities like cardiovascular disease should be actively sought and treated.

6. **Review for modifiable risk factors.** Identify and manage any modifiable risk factors that could be contributing to the worsening dyspnea. Exposure to tobacco smoke or e-cigarettes should be addressed with encouragement and resources for cessation.

7. **Consider stepping up ICS dose (with caution).** If the patient has a low FEV1, especially <60% predicted, a trial of 3 months' treatment with high-dose ICS could be considered, according to the GINA 2024 report. However, the GOLD reports caution against high-dose ICS in patients with features of COPD due to the risk of pneumonia. Therefore, this should be carefully considered alongside the potential benefits and risks.

8.  **Assess for features of asthma-COPD overlap (ACO).** If there are features suggestive of asthma in addition to COPD, the management approach might need to consider both conditions.

9. **Follow-up and reassess.** After any changes in management, the patient should be reassessed in 2-4 weeks to evaluate symptom control and lung function.

In summary, for a COPD patient on ICS monotherapy with increasing dyspnea on walking, the primary management suggestion would be to consider adding a long-acting bronchodilator (LABA or LAMA) or switching to a combination inhaler containing both ICS and LABA. Additionally, referral for pulmonary rehabilitation is strongly recommended. It is also important to reassess the patient's inhaler technique, adherence, lung function, and consider other potential causes of dyspnea and modifiable risk factors. Any changes in treatment should be followed by a timely reassessment.
