# Supplementary material for: Improving Clinical Decision-Making in Treating Airway Diseases With an Expert System Built Upon the Free AI Tool Google NotebookLM
Source: JMIR Med Inform. 2026 Jan 29;14:e78567. doi: 10.2196/78567 (PMC12902755; doi:10.2196/78567)
Supplement: Multimedia Appendix 4 [file medinform_v14i1e78567_app4.docx]

Appendix 4. Unabridged version of Textbox 6

Here's how pulmonary function tests relate to diagnosing airway diseases, and what to do if these tests aren't accessible:

- **COPD Diagnosis:** Spirometry is essential to confirm a COPD diagnosis. The presence of a post-bronchodilator FEV1/FVC ratio of less than 0.7 indicates persistent airflow limitation.
- **Asthma Diagnosis:** Asthma diagnosis relies on a combination of characteristic symptom patterns and evidence of variable expiratory airflow limitation. This should be documented using bronchodilator reversibility testing or other tests.
- **Role of Lung Function Testing:** Lung function testing is essential to confirm persistent expiratory airflow limitation and variable expiratory airflow limitation.
- **Spirometry:** Forced spirometry is the most reproducible and objective measurement of airflow obstruction. It measures forced vital capacity (FVC) and forced expiratory volume in one second (FEV1).
- **When Spirometry Isn't Available for Asthma Diagnosis:**
  - Peak expiratory flow (PEF) can be used, although it is less reliable than spirometry.
  - Assess the patient for a characteristic pattern of respiratory symptoms.
  - Consider a therapeutic trial with SABA and low-dose ICS and evaluate the response.
  - Confirm variable expiratory airflow limitation by PEF. A ≥20% improvement in PEF 15 minutes after administering 2 puffs of albuterol increases the likelihood of diagnosing asthma versus COPD.
  - Improvement in symptoms and PEF after a 4-week therapeutic trial with ICS-containing treatment can help confirm the diagnosis.
- **When Spirometry Isn't Available for COPD Diagnosis:** When routine spirometry is not available, home measurement of peak expiratory flow (PEF) combined with validated patient questionnaires could be used to support or refute a possible diagnosis of COPD.
- **Limitations of PEF:** PEF is not as reliable as spirometry, and a normal PEF does not rule out asthma or COPD. PEF does not correlate well with spirometry results, has low specificity, and cannot differentiate obstructive and restrictive lung function abnormalities.
- **Low-resource Settings:** In low- and middle-income countries (LMICs), access to lung function testing is often very limited. GINA does not recommend that diagnosis should be solely based on syndromic clinical patterns but suggests lung function testing with a PEF meter if spirometry is unavailable.
