# Supplementary material for: Improving Clinical Decision-Making in Treating Airway Diseases With an Expert System Built Upon the Free AI Tool Google NotebookLM
Source: JMIR Med Inform. 2026 Jan 29;14:e78567. doi: 10.2196/78567 (PMC12902755; doi:10.2196/78567)
Supplement: Multimedia Appendix 5 [file medinform_v14i1e78567_app5.docx]

Appendix 5. Unabridged version of Textbox 7.

For your 95-year-old female patient with chronic obstructive pulmonary disease (COPD) and ischemic heart disease (IHD), a **highly individualized, comprehensive, and carefully supervised rehabilitation plan** is crucial, taking into account her advanced age and multiple comorbidities. This plan draws primarily from the Taiwan Pulmonary Rehabilitation Practical Guideline and the Global Initiative for Chronic Obstructive Lung Disease (GOLD) reports.

**I. Overall Approach and Guiding Principles**

1. **Personalized and Integrated Care**: Rehabilitation must be **tailored to her specific needs, capabilities, and comorbidities**, particularly her advanced age (95 years), female gender, and existing Ischemic Heart Disease (IHD). This necessitates a multi-professional healthcare team approach.
2. **Specialist Supervision**: Given her age and IHD, the rehabilitation program should be initiated and conducted under **close medical supervision by specialists**.
3. **Holistic Goals**: The primary goals are to improve her physical and psychological condition, enhance exercise capacity, reduce symptoms, improve quality of life, and promote long-term adherence to health-enhancing behaviors.

**II. Initial Comprehensive Assessment**

Before starting the program, a **thorough baseline assessment** is essential to establish her individual profile and guide the personalized plan. This should include:

- **Patient Goals and Preferences**: Identifying her specific preferences and what she hopes to achieve from rehabilitation.
- **Medical History and Physical Examination**: A detailed history and physical examination are required.
- **Functional Capacity**: Assessment of her exercise capabilities and limitations.
- **Comorbidity Status**: A detailed evaluation of her **Ischemic Heart Disease (IHD)**, including cardiovascular risk, and management according to specific cardiovascular guidelines, irrespective of the COPD.
- **Nutritional Health**: Essential to evaluate, especially given her age and the risk of frailty and muscle loss in COPD patients.
- **Psychological Health**: Assessment of anxiety and depression symptoms, which pulmonary rehabilitation may help reduce.
- **Self-Management Capacity & Health Literacy**: Evaluating her ability to manage her conditions and her understanding of health information.
- **Social Circumstances**: Understanding her social support and living situation.

**III. Rehabilitation Setting and Schedule**

1. **Setting**: For a 95-year-old patient with significant comorbidities, a **supervised setting** is highly recommended, such as an in-patient or out-patient rehabilitation center. **Home-based or tele-rehabilitation** can be considered for improved accessibility and flexibility, especially if she is stable and her condition is not overly complex, and with appropriate remote supervision. Tele-rehabilitation has been shown to improve endurance exercise capacity and is effective in improving clinically relevant outcomes in COPD.
2. **Duration**: Optimal benefits from pulmonary rehabilitation programs are typically achieved within **6 to 8 weeks**. While programs can extend up to 12 weeks, there's no clear evidence of additional benefits beyond 8 weeks.
3. **Frequency**: **Supervised exercise training at least twice weekly** is recommended.

**IV. Components of the Rehabilitation Plan**

1. **Exercise Training**:
   - **Individualized Intensity**: Exercise intensity should be carefully tailored to her capabilities, aiming for **60-80% of her symptom-limited maximum work or heart rate**, or a **Borg-rated dyspnea or fatigue score of 4 to 6 (moderate to severe)**. This must be adjusted cautiously and continuously monitored due to her age and cardiac condition.
   - **Variety of Exercises**: The program should incorporate a combination of different exercise types.
     - **Endurance training**.
     - **Interval training** is a useful strategy if her performance is limited by comorbidities like IHD.
     - **Resistance/strength training** for both upper and lower limbs.
     - Flexibility exercises, inspiratory muscle training, and neuromuscular electrical stimulation can also be included. Walking exercise is also important.
2. **Education and Self-Management**:
   - **Empowerment**: The aim is to motivate and coach her to **positively adapt her health behaviors** and develop skills to better manage her conditions daily.
   - **Written Action Plan**: Provide her with a **written action plan**, customized for her health literacy. This plan should clearly outline how to recognize worsening symptoms related to both IHD (e.g., chest pain, unusual fatigue) and COPD, and what specific actions to take.
   - **Medication Adherence**: Educate her on the critical importance of **medication adherence** for her IHD and COPD, and ensure correct inhaler technique if any inhaled medications are used.
   - **Stress Management**: Provide guidance on **stress management** and help her distinguish between symptoms of anxiety and physical symptoms related to her conditions.
   - **Energy Conservation**: Offer guidance on energy conservation strategies.
3. **Nutritional Support**:
   - Given her age and existing COPD, **nutritional advice and support** are important to counteract potential frailty and muscle loss.
4. **Comorbidity Management**:
   - Her **Ischemic Heart Disease (IHD) must be managed according to specific cardiovascular guidelines**, independent of any respiratory rehabilitation.
   - She should be **monitored for cardiovascular events**, particularly during and for at least 90 days after any acute COPD exacerbations, as there is an increased risk in patients with concomitant IHD.

**V. Follow-up and Monitoring**

- **Regular Review**: **Regular follow-up consultations** by healthcare providers or trained staff are crucial to monitor her progress.
- **Response Assessment**: Her response to the rehabilitation, symptom control, overall health status, and patient satisfaction should be reassessed frequently, with treatment adjustments made as needed.
- **Adherence Check**: Routinely check her adherence to medications and self-management strategies.
- **Spirometry**: Spirometry should be repeated at least annually for COPD patients.

This comprehensive and individualized approach, with diligent monitoring, aims to optimize her physical and psychological well-being, enhancing her ability to manage her conditions and maintain a good quality of life despite her advanced age and complex health profile.
