# Supplementary material for: Improving Clinical Decision-Making in Treating Airway Diseases With an Expert System Built Upon the Free AI Tool Google NotebookLM
Source: JMIR Med Inform. 2026 Jan 29;14:e78567. doi: 10.2196/78567 (PMC12902755; doi:10.2196/78567)
Supplement: Multimedia Appendix 6 [file medinform_v14i1e78567_app6.docx]

Appendix 6. Unabridged version of Textbox 8

The sources discuss future challenges and research directions for airway diseases such as asthma and Chronic Obstructive Pulmonary Disease (COPD).

- **Asthma:**
  - Despite improvements in asthma care and effective medications, many patients worldwide do not benefit from these advances due to inadequate medical facilities and financial resources.
  - Future research should focus on clinical and physiological characteristics, biomarkers, outcomes, and underlying mechanisms in patients with respiratory symptoms or chronic airflow limitation.
  - Further research is needed to understand risk factors for asthma persistence and recurrence, the impact of risk reduction strategies, and whether monitoring after remission improves outcomes.
  - Additional studies could explore whether progression to persistent airflow limitation can be prevented.
- **COPD:**
  - More research is needed to guide better recognition and safe, effective treatment of COPD.
  - There's a need to study clinical and physiological characteristics, biomarkers, outcomes and underlying mechanisms in broad populations of patients with respiratory symptoms or with chronic airflow limitation.
  - Future studies should focus on defining phenotypes and mechanisms to ensure the safety of pharmacological treatments.
  - Research is necessary to determine the best treatment for individuals with 'Pre-COPD' or PRISm (Preserved Ratio Impaired Spirometry) who are at risk of developing airflow obstruction.
  - Further research is required to ascertain the direction(s) of causality and possible prognostic, diagnostic, or therapeutic applications of dysbiosis in COPD.
- **General points on both Asthma and COPD:**
  - Implementation of evidence-based recommendations requires an evidence-based strategy involving professional groups and stakeholders, while considering local cultural and socioeconomic conditions.
  - There is a need for evidence-based definitions and a more detailed classification of patients who present overlapping features of asthma and COPD, and to encourage the development of specific interventions for clinical use.
  - It is important to consider the patient's goals, beliefs, and concerns about asthma and medications.
  - Practical issues such as inhaler availability, technique, adherence, and cost should be addressed.
  - Treatment of modifiable risk factors, comorbidities, and non-pharmacological strategies should be considered.
  - The initial management of asthma and COPD can be carried out at primary care level, but referral for further diagnostic procedures may be necessary, especially for patients with features of both asthma and COPD.
