# Supplementary material for: Improving Clinical Decision-Making in Treating Airway Diseases With an Expert System Built Upon the Free AI Tool Google NotebookLM
Source: JMIR Med Inform. 2026 Jan 29;14:e78567. doi: 10.2196/78567 (PMC12902755; doi:10.2196/78567)
Supplement: Multimedia Appendix 7 [file medinform_v14i1e78567_app7.docx]

Appendix 7. Unabridged version of Textbox 9.

In the past five years, pharmacological treatment for both Chronic Obstructive Pulmonary Disease (COPD) and asthma has seen a clear trend towards more individualized, exacerbation-focused, and strategically combined therapies, with a strong emphasis on proper inhaler use and minimizing oral corticosteroid exposure.

**Overarching Trends for Both COPD and Asthma (Past 5 Years)**

1. **Individualized and Patient-Centered Care:** Both Global Initiative for Chronic Obstructive Lung Disease (GOLD) and Global Initiative for Asthma (GINA) guidelines advocate for **individualized treatment regimens** guided by the severity of symptoms, risk of exacerbations, potential side effects, comorbidities, drug availability, cost, and the patient's response, preferences, and ability to use various drug delivery devices.
2. **Focus on Exacerbation Reduction:** A primary goal across both conditions is to **reduce the frequency and severity of exacerbations**, as these significantly impact patient quality of life and mortality.
3. **Emphasis on Inhaler Technique and Adherence:** Regular assessment and improvement of **inhaler technique** and **medication adherence** are consistently highlighted as critical for effective treatment, with digital monitoring tools gaining recognition.
4. **Minimizing Oral Corticosteroid (OCS) Use:** Both guidelines strongly recommend optimizing inhaled therapy to reduce the need for OCS due to their significant systemic side effects.

**Trends in COPD Pharmacological Treatment (Past 5 Years)**

The GOLD reports (2021, 2022, 2023, 2024, 2025) and the 2023 Taiwan COPD Clinical Care Guidelines emphasize these trends:

1. **Long-Acting Bronchodilators (LABA and/or LAMA) as First-Line:** **Pharmacotherapy for stable COPD primarily begins with long-acting bronchodilators** to reduce symptoms, the frequency and severity of exacerbations, and to improve exercise tolerance and health status.
2. **Strategic Use of Inhaled Corticosteroids (ICS):**
   - ICS **should not be used alone as monotherapy** in COPD.
   - ICS are recommended as an **add-on to bronchodilator therapy for specific patient populations**. These include patients with a history of hospitalizations for COPD, ≥2 exacerbations per year requiring oral corticosteroids, or **blood eosinophil counts ≥300/µL**, or a history of asthma or concomitant asthma.
   - **High-dose ICS should be avoided** in patients with features of COPD due to the increased risk of pneumonia.
3. **Increasing Role of Triple Therapy (LABA+LAMA+ICS):** There is growing evidence and recognition of fixed-dose inhaled triple combinations. Recent randomized controlled trials (IMPACT and ETHOS) provide new evidence on **mortality reduction** with these combinations compared to dual bronchodilation, especially in symptomatic patients with a history of frequent and/or severe exacerbations who were previously on maintenance therapy.
4. **Phosphodiesterase-4 (PDE4) Inhibitors (Roflumilast):** Roflumilast remains an option for reducing moderate and severe exacerbations, particularly in patients with chronic bronchitis, severe to very severe COPD, and a history of exacerbations. Its benefits are reported to be greater in patients with a prior history of hospitalization for acute exacerbations.
5. **New Treatment Inclusions:** The **GOLD 2025 report explicitly includes new treatments, ensifentrine and dupilumab**, in figures for maintenance medications, bronchodilators, anti-inflammatory therapy, and interventions to reduce exacerbations, indicating an expansion of therapeutic options. Dupilumab is also noted for COPD with Type 2 inflammation indicated by eosinophil counts.
6. **Oral Glucocorticoids:** Oral glucocorticoids have **no role in the chronic daily treatment of COPD** due to their lack of benefit compared to a high rate of systemic complications, though they are used for acute exacerbations.
7. **Prophylactic Antibiotics:** While older studies showed no effect, later studies suggest that regular use of some antibiotics (e.g., azithromycin or erythromycin for one year) may reduce the exacerbation rate in patients prone to exacerbations, though with considerations for side effects and bacterial resistance.

**Trends in Asthma Pharmacological Treatment (Past 5 Years)**

GINA reports (2021, 2022, 2023, 2024, 2025) and the 2022 Taiwan Adult Asthma Clinical Care Guidelines highlight significant shifts:

1. **Shift Away from SABA Monotherapy:** A **fundamental change** since GINA 2019 is that **treatment of asthma with short-acting beta2-agonists (SABA) alone is no longer recommended** for adults and adolescents, as it is associated with an increased risk of severe exacerbations and mortality. Overuse of SABA is also identified as an independent risk factor for mortality.
2. **Introduction of Two Treatment Tracks (GINA):** GINA has clarified treatment options into two "treatment tracks" for adults and adolescents:
   - **Track 1 (Preferred):** This approach recommends **as-needed low-dose ICS-formoterol as the reliever** (Anti-Inflammatory Reliever, AIR therapy). This track is preferred because it significantly **reduces the risk of severe exacerbations** compared with SABA-based regimens, provides similar symptom control, and offers a simpler regimen.
   - **Track 2 (Alternative):** Involves daily maintenance ICS (or ICS-LABA in later steps) combined with as-needed SABA or, in some cases, as-needed ICS-SABA for relief.
3. **ICS as the Cornerstone of Controller Therapy:** **Inhaled corticosteroids (ICS) remain the basis of pharmacotherapy for asthma** to reduce airway inflammation, control symptoms, and reduce the risk of severe exacerbations and death.
4. **LABA and LAMA as Add-on Therapy (Never Monotherapy):** Long-acting bronchodilators (LABA and/or LAMA) are used as **add-on treatments to ICS** when required for symptom control but are **contraindicated as monotherapy in asthma** due to the risk of severe exacerbations and death. LAMA (e.g., tiotropium by mist inhaler) is an add-on option at Step 5 (or non-preferred Step 4) for patients with uncontrolled asthma despite ICS-LABA, providing modest improvements in lung function and small reductions in exacerbations. Combination ICS-LABA-LAMA inhalers are available for adults ≥18 years.
5. **Expansion of Biologic Therapies:** For severe asthma (Step 5), there is an increasing use of **add-on biologic therapies**. Dupilumab, an anti-IL4R antibody, is mentioned as an option for children aged 6-11 years in the 2022 GINA report.
6. **Discontinuation of Certain Medications:**
   - **Fenoterol** has been added to the list of non-recommended bronchodilators due to its higher risk of cardiovascular adverse effects and asthma mortality.
   - **Chromone pressurized metered dose inhalers** have been globally discontinued due to their lack of efficacy compared to even low-dose ICS and burdensome maintenance requirements.
