# Supplementary material for: Improving Clinical Decision-Making in Treating Airway Diseases With an Expert System Built Upon the Free AI Tool Google NotebookLM
Source: JMIR Med Inform. 2026 Jan 29;14:e78567. doi: 10.2196/78567 (PMC12902755; doi:10.2196/78567)
Supplement: Multimedia Appendix 8 [file medinform_v14i1e78567_app8.docx]

Appendix 8. Unabridged version of Textbox 10.

For further readings about the treatment of intractable asthma, which is often referred to in the sources as **severe asthma** or **difficult-to-treat asthma**, you may find the following publications insightful. These sources provide detailed information on diagnosis, management, and treatment options for patients whose asthma remains uncontrolled despite standard therapies:

**Comprehensive Guidelines and Reviews:**

- **Global Strategy for Asthma Management and Prevention (GINA)**: The GINA reports are frequently referenced for their comprehensive guidance on asthma management. Specifically, the sections within these reports on "Difficult-to-treat and severe asthma" provide a roadmap for assessment and management. You can refer to the detailed sections in the provided GINA reports, such as:
  - **GINA-2023-Report**: Includes indications for referral for expert advice and updated guidance on managing severe asthma, emphasizing that biologic therapy is recommended only if asthma is severe and existing treatment has been optimized. It also covers investigating patients with poor symptom control and/or exacerbations despite treatment.
  - **GINA-2024-strategy-report**: Provides updated guidance on managing severe asthma, including clarification on referring patients to a specialist center for phenotypic assessment and consideration of add-on therapy.
  - **GINA-2025-Clean**: Features sections on investigating and managing difficult-to-treat asthma, including confirming diagnosis, optimizing treatment, and considering additional diagnostic investigations and add-on therapies.
  - **GINA-Main-Report-2021-V2**: Contains a guide and decision tree on the diagnosis and management of difficult-to-treat and severe asthma in adolescent and adult patients.
  - **GINA-Main-Report-2022**: Discusses management of acute asthma in healthcare settings, noting that more studies with ICS-formoterol are needed for primary care and emergency departments. It also includes information on optimizing management for difficult-to-treat asthma.
  - **GINA-Severe-Asthma-Guide-2023-WEB**: Specifically details the investigation and management of difficult-to-treat asthma in adults and adolescents, including optimizing management, assessing severe asthma phenotypes, and considering add-on biologic therapies.
- **International ERS/ATS guidelines on definition, evaluation and treatment of severe asthma**: This guideline [1] provides a comprehensive definition, evaluation, and treatment approach for severe asthma.
- **Severe and difficult-to-treat asthma in adults**: For a focused review, consider the article by **Israel E, Reddel HK** [2].
- **Current treatment of severe asthma**: **Hashimoto S and Bel EH** [3] provide insights into current treatment strategies for severe asthma.
- **The prevalence of severe refractory asthma**: The study by **Hekking P et al.** [4] specifically addresses the prevalence of severe refractory asthma .

**Pharmacological Interventions:**

- **Biologic Therapies**:
  - **EAACI Biologicals Guidelines-Recommendations for severe asthma**: **Agache I et al.** [5] offer guidelines and recommendations for biologics in severe asthma.
  - **Tezepelumab**: For adults and adolescents with severe, uncontrolled asthma, **Menzies-Gow A et al.** (2021) [6] and **Corren J et al.** (2017) [7] discuss the efficacy and safety of tezepelumab . Further evaluation of its oral corticosteroid-sparing effect can be found in **Wechsler ME et al.** (2022)[8].
  - **Dupilumab**: Its efficacy and safety in uncontrolled moderate-to-severe asthma, including in children and adults requiring maintenance oral corticosteroids (OCS), is covered by **Bacharier LB et al.** (2021)[9], **Castro M et al.** (2018)[10], and **Wenzel S et al.** (2016)[11].
  - **Benralizumab**: Its oral glucocorticoid-sparing effect in eosinophilic asthma is explored by **Nair P et al.** (2017)[12].
  - **Mepolizumab**: Long-term safety and durability of clinical response in severe eosinophilic asthma is assessed by **Khatri S et al.** (2019)[13], and the impact of discontinuing mepolizumab is discussed by **Moore WC et al.** (2022)[14].
- **Add-on treatments (non-biologic)**:
  - **Tiotropium**: **Bel EH** [15] discusses the promise and caution associated with tiotropium for asthma. For school-age children with moderate-to-severe symptomatic asthma, **Rodrigo GJ and Neffen H** [16] provide a systematic review.
  - **Azithromycin**: **Brusselle GG et al.** (2013) [17] and **Gibson PG et al.** (2017) [18] investigate azithromycin for the prevention of exacerbations in severe asthma and its effect on quality of life in adults with persistent uncontrolled asthma . **Taylor SL et al.** (2019) [19] examined its long-term effects on *Haemophilus influenzae* and antibiotic resistance.
  - **Oral Corticosteroids (OCS)**: The sources highlight that OCS should be considered only as a last resort due to serious long-term side-effects. **Lefebvre P et al.** (2015)[20], **Bleecker ER et al.** (2020)[21], and **Walsh LJ et al.** (2001)[22] provide literature reviews on acute and chronic systemic corticosteroid-related complications .

**Diagnostic and Management Strategies:**

- **Sputum-guided treatment**: **Petsky HL et al.** (2017)[23] discuss tailored interventions based on sputum eosinophils versus clinical symptoms for asthma in children and adults, showing a reduced risk of exacerbations in adults with moderate-severe asthma.
- **Bronchial Thermoplasty**: **Chaudhuri R et al.** (2021)[24] report on the safety and effectiveness of bronchial thermoplasty after 10 years in patients with persistent asthma. **Chupp G et al.** (2017)[25] describe the long-term outcomes of bronchial thermoplasty.
- **Importance of Adherence and Inhaler Technique**:
  - **McNicholl DM et al.** (2012)[26] found that fractional exhaled nitric oxide (FeNO) suppression can help identify nonadherence in difficult asthma.
  - **Gamble J et al.** (2009)[27] investigated the prevalence of nonadherence in difficult asthma .
  - **Sulaiman I et al.** (2018)[28] conducted a trial on feedback for inhaler adherence and technique in severe uncontrolled asthma.

These readings should provide a comprehensive overview of the current understanding and treatment approaches for intractable asthma.

References

[1] Chung KF, Wenzel SE, Brozek JL, et al. International ERS/ATS guidelines on definition, evaluation and treatment of severe asthma. *Eur Respir J*. 2014;43(2):343-373. doi:10.1183/09031936.00202013

[2] Israel E, Reddel HK. Severe and Difficult-to-Treat Asthma in Adults. *N Engl J Med*. 2017;377(10):965-976. doi:10.1056/NEJMra1608969

[3] Hashimoto S, Bel EH. Current treatment of severe asthma. *Clin Exp Allergy*. 2012;42(5):693-705. doi:10.1111/j.1365-2222.2011.03936.x

[4] Hekking PW, Wener RR, Amelink M, Zwinderman AH, Bouvy ML, Bel EH. The prevalence of severe refractory asthma. *J Allergy Clin Immunol*. 2015;135(4):896-902. doi:10.1016/j.jaci.2014.08.042

[5] Agache I, Akdis CA, Akdis M, et al. EAACI Biologicals Guidelines-Recommendations for severe asthma. *Allergy*. 2021;76(1):14-44. doi:10.1111/all.14425

[6] Menzies-Gow A, Corren J, Bourdin A, et al. Tezepelumab in Adults and Adolescents with Severe, Uncontrolled Asthma. *N Engl J Med*. 2021;384(19):1800-1809. doi:10.1056/NEJMoa2034975

[7] Corren J, Parnes JR, Wang L, et al. Tezepelumab in Adults with Uncontrolled Asthma. *N Engl J Med*. 2017;377(10):936-946. doi:10.1056/NEJMoa1704064

[8] Wechsler ME, Menzies-Gow A, Brightling CE, et al. Evaluation of the oral corticosteroid-sparing effect of tezepelumab in adults with oral corticosteroid-dependent asthma (SOURCE): a randomised, placebo-controlled, phase 3 study. *Lancet Respir Med*. 2022;10(7):650-660. doi:10.1016/S2213-2600(21)00537-3

[9] Bacharier LB, Maspero JF, Katelaris CH, et al. Dupilumab in Children with Uncontrolled Moderate-to-Severe Asthma. *N Engl J Med*. 2021;385(24):2230-2240. doi:10.1056/NEJMoa2106567

[10] Castro M, Corren J, Pavord ID, et al. Dupilumab Efficacy and Safety in Moderate-to-Severe Uncontrolled Asthma. *N Engl J Med*. 2018;378(26):2486-2496. doi:10.1056/NEJMoa1804092

[11] Wenzel S, Castro M, Corren J, et al. Dupilumab efficacy and safety in adults with uncontrolled persistent asthma despite use of medium-to-high-dose inhaled corticosteroids plus a long-acting β2 agonist: a randomised double-blind placebo-controlled pivotal phase 2b dose-ranging trial. *Lancet*. 2016;388(10039):31-44. doi:10.1016/S0140-6736(16)30307-5

[12] Nair P, Wenzel S, Rabe KF, et al. Oral Glucocorticoid-Sparing Effect of Benralizumab in Severe Asthma. *N Engl J Med*. 2017;376(25):2448-2458. doi:10.1056/NEJMoa1703501

[13] Khatri S, Moore W, Gibson PG, et al. Assessment of the long-term safety of mepolizumab and durability of clinical response in patients with severe eosinophilic asthma. *J Allergy Clin Immunol*. 2019;143(5):1742-1751.e7. doi:10.1016/j.jaci.2018.09.033

[14] Moore WC, Kornmann O, Humbert M, et al. Stopping *versus* continuing long-term mepolizumab treatment in severe eosinophilic asthma (COMET study). *Eur Respir J*. 2022;59(1):2100396. Published 2022 Jan 6. doi:10.1183/13993003.00396-2021

[15] Bel EH. Tiotropium for asthma--promise and caution. *N Engl J Med*. 2012;367(13):1257-1259. doi:10.1056/NEJMe1209381

[16] Rodrigo GJ, Neffen H. A Systematic Review of the Efficacy and Safety of a Fixed-Dose Combination of Umeclidinium and Vilanterol for the Treatment of COPD. *Chest*. 2015;148(2):397-407. doi:10.1378/chest.15-0084

[17] Brusselle GG, Vanderstichele C, Jordens P, et al. Azithromycin for prevention of exacerbations in severe asthma (AZISAST): a multicentre randomised double-blind placebo-controlled trial. *Thorax*. 2013;68(4):322-329. doi:10.1136/thoraxjnl-2012-202698

[18] Gibson PG, Yang IA, Upham JW, et al. Effect of azithromycin on asthma exacerbations and quality of life in adults with persistent uncontrolled asthma (AMAZES): a randomised, double-blind, placebo-controlled trial. *Lancet*. 2017;390(10095):659-668. doi:10.1016/S0140-6736(17)31281-3

[19] Taylor SL, Leong LEX, Mobegi FM, et al. Long-Term Azithromycin Reduces *Haemophilus influenzae* and Increases Antibiotic Resistance in Severe Asthma. *Am J Respir Crit Care Med*. 2019;200(3):309-317. doi:10.1164/rccm.201809-1739OC

[20] Lefebvre P, Duh MS, Lafeuille MH, et al. Acute and chronic systemic corticosteroid-related complications in patients with severe asthma. *J Allergy Clin Immunol*. 2015;136(6):1488-1495. doi:10.1016/j.jaci.2015.07.046

[21] Bleecker ER, Menzies-Gow AN, Price DB, et al. Systematic Literature Review of Systemic Corticosteroid Use for Asthma Management. *Am J Respir Crit Care Med*. 2020;201(3):276-293. doi:10.1164/rccm.201904-0903SO

[22] Walsh LJ, Wong CA, Oborne J, et al. Adverse effects of oral corticosteroids in relation to dose in patients with lung disease. *Thorax*. 2001;56(4):279-284. doi:10.1136/thorax.56.4.279

[23] Petsky HL, Li A, Chang AB. Tailored interventions based on sputum eosinophils versus clinical symptoms for asthma in children and adults. *Cochrane Database Syst Rev*. 2017;8(8):CD005603. Published 2017 Aug 24. doi:10.1002/14651858.CD005603.pub3

[24] Chaudhuri R, Rubin A, Sumino K, et al. Safety and effectiveness of bronchial thermoplasty after 10 years in patients with persistent asthma (BT10+): a follow-up of three randomised controlled trials. *Lancet Respir Med*. 2021;9(5):457-466. doi:10.1016/S2213-2600(20)30408-2

[25] Chupp G, Laviolette M, Cohn L, et al. Long-term outcomes of bronchial thermoplasty in subjects with severe asthma: a comparison of 3-year follow-up results from two prospective multicentre studies. *Eur Respir J*. 2017;50(2):1700017. Published 2017 Aug 31. doi:10.1183/13993003.00017-2017

[26] McNicholl DM, Stevenson M, McGarvey LP, Heaney LG. The utility of fractional exhaled nitric oxide suppression in the identification of nonadherence in difficult asthma. *Am J Respir Crit Care Med*. 2012;186(11):1102-1108. doi:10.1164/rccm.201204-0587OC

[27] Gamble J, Stevenson M, McClean E, Heaney LG. The prevalence of nonadherence in difficult asthma. *Am J Respir Crit Care Med*. 2009;180(9):817-822. doi:10.1164/rccm.200902-0166OC

[28] Sulaiman I, Greene G, MacHale E, et al. A randomised clinical trial of feedback on inhaler adherence and technique in patients with severe uncontrolled asthma. *Eur Respir J*. 2018;51(1):1701126. Published 2018 Jan 4. doi:10.1183/13993003.01126-2017
