# Supplementary material for: Improving Clinical Decision-Making in Treating Airway Diseases With an Expert System Built Upon the Free AI Tool Google NotebookLM
Source: JMIR Med Inform. 2026 Jan 29;14:e78567. doi: 10.2196/78567 (PMC12902755; doi:10.2196/78567)
Supplement: Multimedia Appendix 9 [file medinform_v14i1e78567_app9.docx]

Appendix 9. Unabridged version of Toolbox 12

Here is a simulated patient case with asthma- or COPD-like symptoms, including their pulmonary function values, designed to test your diagnostic skills. Following the case, the diagnosis, reasons, and treatment suggestions, supported by the provided sources, will be given.

--------------------------------------------------------------------------------

**Simulated Patient: Mr. David Lee**

**Patient Background:** Mr. David Lee is a 58-year-old male with a **25 pack-year smoking history**, though he states he is currently trying to quit. He reports a **chronic cough** with some **mucus production** for the past 8 years. He also experiences **intermittent shortness of breath** on exertion, particularly when climbing stairs. Lately, he's noticed that his **breathing feels tighter in the mornings**, and he occasionally **hears a whistling sound (wheeze)** when breathing out, especially after exposure to cold air. These wheezing episodes are variable in intensity, and he notes that his symptoms sometimes improve with a relative's "asthma inhaler" (a short-acting beta2-agonist, SABA).

**Symptoms:**

• **Chronic cough** with **mucus production** (8 years duration).

• **Intermittent dyspnea** on exertion (e.g., climbing stairs).

• **Morning chest tightness**.

• **Occasional expiratory wheezing**, variable in intensity, exacerbated by **cold air**.

• Reports temporary improvement with SABA.

• Significant **smoking history** (25 pack-years).

**Pulmonary Function Values:**

• **Pre-Bronchodilator Spirometry:**

    ◦ Forced Expiratory Volume in 1 second (FEV1): 60% of predicted

    ◦ Forced Vital Capacity (FVC): 80% of predicted

    ◦ FEV1/FVC Ratio: **65%** (indicating airflow limitation)

• **Post-Bronchodilator Spirometry** (after administering 400 mcg of salbutamol, a SABA):

    ◦ FEV1: 75% of predicted (representing an **increase of 15% and 400 mL from baseline**).

    ◦ FVC: 85% of predicted

    ◦ FEV1/FVC Ratio: **80%** (post-BD ratio is now within the normal range, indicating reversibility)

• **Diurnal Peak Expiratory Flow (PEF) Monitoring** (averaged over two weeks, performed at home prior to this visit):

    ◦ Average daily diurnal PEF variability: **18%**.

--------------------------------------------------------------------------------

**Diagnosis, Reasons, and Treatment Suggestion for Mr. David Lee**

**Diagnosis:** Based on the clinical presentation and pulmonary function values, Mr. David Lee's most likely diagnosis is **Asthma, with features that overlap with Chronic Obstructive Pulmonary Disease (COPD)**, often referred to as **Asthma-COPD Overlap (ACO)**. Given the *full reversibility* of his FEV1/FVC ratio and the *significant* bronchodilator response, the primary clinical approach should focus on asthma management.

**Reasons for Diagnosis:**

1. **Characteristic Variable Respiratory Symptoms:** Mr. Lee presents with a history of **variable respiratory symptoms**, including chronic cough, intermittent dyspnea on exertion, morning chest tightness, and occasional wheezing exacerbated by cold air. These symptoms also improve with a SABA. The variability of symptoms over time and in intensity, and their triggers, are classic features that strongly support an asthma diagnosis. In adults, isolated cough is seldom due to asthma, but his presentation includes multiple symptoms.

2. **Pulmonary Function Test (PFT) Findings Indicating Variable Expiratory Airflow Limitation:**

    ◦ **Bronchodilator Responsiveness:** His pre-bronchodilator FEV1/FVC ratio of 65% initially shows expiratory airflow limitation. However, after administering salbutamol, his FEV1 increased by **15% and 400 mL from baseline**, and his FEV1/FVC ratio normalized to **80%**. According to guidelines, an increase in FEV1 of **>12% and >200 mL** (with **greater confidence if >15% and >400 mL**) after bronchodilator administration is a key criterion for confirming variable expiratory airflow limitation characteristic of asthma. The normalization of his FEV1/FVC ratio (above the 0.70 threshold) confirms a fully reversible obstruction. This significant reversibility makes asthma the predominant diagnosis, despite the initial obstructive pattern.

    ◦ **Excessive PEF Variability:** His average daily diurnal PEF variability of **18%** over two weeks is considered excessive for adults (typically >10% for adults or >13% for children, depending on the guideline version used). This excessive variation in lung function is a strong indicator of asthma and suggests suboptimal asthma control.

3. **Smoking History and Overlap Features:** Mr. Lee's 25 pack-year smoking history and chronic productive cough are risk factors and symptoms typically associated with COPD. However, the clear evidence of variable expiratory airflow limitation with significant bronchodilator responsiveness outweighs a pure COPD diagnosis. When a patient presents with features of both asthma and COPD, guidelines recommend **treating the patient as asthma** to reduce the risk of severe exacerbations and death. It is crucial to assess patients with a >10 pack-year smoking history for COPD or for asthma+COPD.

**Treatment Suggestion:** For Mr. Lee, the treatment strategy should prioritize asthma management due to the clear evidence of reversible airflow limitation and variable symptoms. Simultaneously, his smoking history and the associated risks must be addressed.

1. **Smoking Cessation:** This is the most crucial intervention for any patient with a smoking history and airway disease. He should receive strong encouragement and resources for smoking cessation.

2. **Inhaled Corticosteroid (ICS)-containing therapy:** ICS are essential for asthma, either alone or in combination with a long-acting beta2-agonist (LABA), to reduce the risk of severe exacerbations, prevent decline in lung function, and improve symptom control. Given his symptoms and PFTs, **regular ICS-containing controller treatment is strongly indicated**.

3. **Reliever Medication:**

    ◦ An **anti-inflammatory reliever (AIR)**, such as low-dose ICS-formoterol, taken as needed for symptoms and before exercise or allergen exposure, is the preferred approach as it reduces the risk of severe exacerbations and urgent healthcare utilization compared to short-acting beta2-agonists (SABA) alone.

    ◦ If AIR is not chosen or available, a **SABA** (like salbutamol) can be used as a reliever, but it should **not be used alone without ICS**.

4. **Follow-up and Monitoring:**

    ◦ **Regular monitoring** of his symptoms (e.g., using an Asthma Control Test or Asthma Control Questionnaire) and lung function (FEV1 and PEF variability) is crucial to assess treatment response and adjust therapy as needed.

    ◦ Lung function should be recorded at diagnosis, 3–6 months after starting treatment (to establish his personal best FEV1), and periodically thereafter (e.g., every 1-2 years for most adults, more frequently for higher-risk patients) for ongoing risk assessment.

    ◦ PEF monitoring at home can be useful for short-term assessment of treatment response, evaluating triggers, and establishing a baseline for action plans.

5. **Comorbidities and Differential Diagnosis:** Although the PFTs strongly point to asthma, his smoking history necessitates careful consideration of other potential comorbidities. Symptoms of dyspnea, cough, and wheeze can also be due to conditions like COPD, obesity, cardiac disease, or deconditioning. Regular assessment for these conditions is recommended.
